# Correlation Between Hypothyroidism During Pregnancy and Glucose and Lipid Metabolism in Pregnant Women and Its Influence on Pregnancy Outcome and Fetal Growth and Development

**DOI:** 10.3389/fsurg.2022.863286

**Published:** 2022-03-28

**Authors:** Da Xu, Haolin Zhong

**Affiliations:** ^1^Department of Endocrinology, Zhuji People's Hospital, Zhuji, China; ^2^Department of Obstetrics, Zhuji Maternal and Child Health Care Hospital, Zhuji, China

**Keywords:** hypothyroidism during pregnancy, glucose metabolism, lipid metabolism, pregnancy outcome, fetal growth and development

## Abstract

**Purpose:**

To observe the correlation between hypothyroidism during pregnancy and glucose and lipid metabolism in pregnant women and its influence on a pregnancy outcome and fetal growth and development.

**Methods:**

About 152 patients with hypothyroidism during pregnancy in our hospital from June 2017 to June 2020 were selected as the observation group and divided into the overt hypothyroidism (OH) group, the subclinical hypothyroidism (SCH) group, and the low T_4_ group. Another 60 pregnant women with normal antenatal examination and normal thyroid function were selected as the normal group. The glucose and lipid metabolism indexes of each group were compared. The pregnant women in the OH group and the SCH group were given levothyroxine intervention, and the pregnancy outcome and infant development of the two groups were compared.

**Results:**

The fasting blood glucose and hemoglobin A1c, triglyceride and low-density lipoprotein of the OH group and the SCH group were higher than the low T_4_ group and the normal group, and the OH group was higher than the SCH group (*p* < 0.05). The incidence of premature delivery and premature rupture of membranes at term (PROM at term) in the hypothyroidism non-control group was higher than the hypothyroidism control group (*p* < 0.05). The mental development index and the psychomotor development index in the hypothyroidism non-control group were lower than the hypothyroidism control group (*p* < 0.05).

**Conclusion:**

Pregnant women with hypothyroidism during pregnancy are more prone to glucose and lipid metabolism disorder, which increases the risk of premature delivery and PROM at term, and has certain influence on the intellectual development and psychomotor development of infants.

## Introduction

Thyroid dysfunction is one of the common endocrine complications in pregnancy, especially hypothyroidism in pregnancy. Thyroid hormone (TH) is the most important endocrine hormone in the body, which can promote the synthesis of protein, RNA, DNA, and special enzymes in fetal tissues and cells, TH can regulate the metabolism of carbohydrates, calcium, phosphorus, fat, and other energy substances in pregnant women and fetus, and promote the growth and development of fetal bones and reproductive organs, and is very important to maintain the normal development and maturity of fetus (1, 2). During pregnancy, the hypothalamus-pituitary-thyroid regulatory system of pregnant women is in a stress state, and, during pregnancy, it is in a special endocrine state, which leads to the decrease of TH synthesis and the defect of thyroid receptor function, resulting in the decrease of the utilization rate of TH (3). Hypothyroidism can lead to a series of related clinical symptoms such as hypometabolism in pregnant women, with listlessness, fatigue, lethargy, pale face, rough skin, and decreased heart rate as the main manifestations (4). The incidence of hypothyroidism is high among women of childbearing age. The incidence of overt hypothyroidism (OH) in pregnant women is 1–2%, that of subclinical hypothyroidism (SCH) is 2–5%, and that of isolated low T_4_ is 8–10% (5). Generally speaking, hypothyroidism is often ignored by people because the onset of hypothyroidism is hidden. In recent years, some scholars have suggested that hypothyroidism during pregnancy may affect maternal glucose and lipid metabolism and offspring development, which seriously endangers maternal and infant health (6). In this study, we observed the glucose and lipid metabolism and a pregnancy outcome of pregnant women with hypothyroidism during pregnancy, and followed up the fetus in order to improve the clinical outcome of pregnant women and fetus.

## Materials and Methods

### Object

About 152 patients with hypothyroidism during pregnancy in our hospital from June 2017 to June 2020 were selected as the observation group. Inclusion criteria: age > 18 years old; the first gestational week was <28 weeks; single pregnancy; the patient had a formal birth examination in the department of obstetrics of our hospital and delivered in our hospital. Exclusion criteria: a history of thyroid disease before pregnancy; pre-pregnancy with abnormal glucose and lipid metabolism-related diseases; in the past 3 months, patient has taken drugs that affect thyroid hormones or glucose and lipid metabolism indexes; the fetus was lost before 28 weeks of gestation; complicated with serious organic diseases; lost or dropped out of the study. Another 60 pregnant women with normal antenatal examination and normal thyroid function were selected as the normal group. All the subjects were informed and agreed, and this study was reviewed by the ethics committee.

### Research Methods

In the second trimester of pregnancy (14–27^+6^ weeks of pregnancy), in the morning, 3 ml of fasting venous blood was collected from all the subjects, and the blood was centrifuged at 3,500 r/5 min for 5 min at room temperature, and the serum was separated. The levels of serum-free triiodothyronine (FT_3_), free tetraiodothyronine (FT_4_), and thyroid-stimulating hormone (TSH) were detected by an automatic chemiluminescence instrument. Fasting blood glucose (FBG) and hemoglobin A1c (HbA1c) were measured. Triglyceride (TG), total cholesterol (TC), high-density lipoprotein (HDL), and low-density lipoprotein (LDL) were measured.

According to the diagnostic criteria of hypothyroidism during pregnancy (7), patients with hypothyroidism were divided into: the (1) OH group: serum TSH > 3.6 mIU/L and FT_4_ decreased, or serum TSH > 10 mIU/L regardless of whether FT_4_ was normal or not; the (2) SCH group: serum TSH > 3.6 mIU/L, the serum FT_4_ level was normal; the (3) Low T_4_ group: the TSH level was normal, but the serum FT_4_ level was lower than normal.

From the date of the diagnosis, the pregnant women with SCH and OH were treated with levothyroxine sodium tablets (specification: 50 ug), and FT_3_, FT_4_, and TSH were detected every 4 weeks. The therapeutic target of serum TSH was: 2–3. mIU/L in the second trimester. The dosage of levothyroxine sodium tablets had large individual variability, which required clinicians to evaluate factors, such as the cause of hypothyroidism, pre-pregnancy TSH levels, and other factors before treatment, and adjust the dosage according to individual circumstances. Pregnant women with hypothyroidism will not be treated; only FT_3_, FT_4_, and TSH will be detected every month, and then treated if the disease meets the requirements of SCH or OH. According to the level of serum TSH of the pregnant women before labor, they were divided into the hypothyroidism control group, and the serum TSH was kept in the target range through treatment; in the uncontrolled hypothyroidism group, the serum TSH was not controlled within the target range due to various reasons, such as the pregnant women's disobedience to the doctor's advice and refusal for treatment.

### Evaluation Methods

All the subjects were followed up until delivery, and adverse pregnancy outcomes, such as premature delivery (delivery between >28 weeks and <37 weeks of gestation), abortion (termination of pregnancy due to pregnancy <28 weeks and fetal weight < 1,000 g), premature rupture of membranes at term (PROM at term) (After 37 weeks of gestation, the membranes rupture naturally), infants of low-birth weight (fetal birth weight < 2,500 g), and fetal distress (fetal heart rate, <120 beats/min or >160 beats/min on auscultation test) were recorded.

Within 18 months of live births, infants' intelligence development and psychomotor development were measured by Bayley scales of infant development (BSID) (revised edition of Chinese cities), and mental development index (MDI) and psychomotor development index (PDI) were calculated (8). MDI was used to test the infant's response to stimuli, hand-eye coordination, language, exploratory activities, cognitive ability, etc.; PDI was used to test the gross motor and fine motor of all parts of the infant's body. MDI included 163 items and PDI included 81 items. The larger the index, the better the infant's development. The test results show that the developmental quotient ≤ 69 was diagnosed as developmental retardation. The infants were tested in a testing room by specially trained testers.

### Statistical Methods

SPSS22.0 software was used for analysis, and the measured data were expressed by $\bar{x}$ ± s and compared by *t*-test. The counting data were expressed as % and compared by χ^2^ test. *p* < 0.05, the difference was statistically significant.

## Results

### Incidence of Hypothyroidism During Pregnancy

There were 152 cases in the observation group, including 56 pregnant women with OH, 53 pregnant women with SCH, and 43 cases with low T_4_. During the study period, no pregnant women with low T_4_ changed into SCH or OH. After the treatment, hypothyroidism was controlled in 85 cases and uncontrolled in 24 cases among the 109 pregnant women with SCH or OH.

### Comparison of Blood Glucose Levels in Each Group

**Figure 1:**
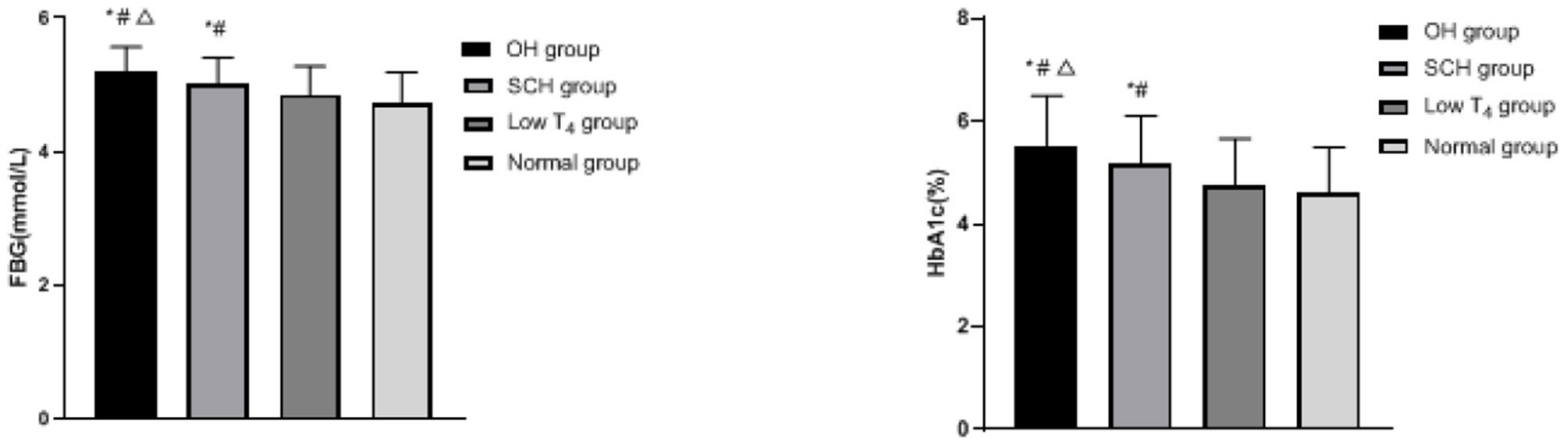
Comparison of blood glucose levels in each group; compared with the normal group, ^*^p < 0.05; compared with the low T_4_ group, ^#^*p* < 0.05; compared with the SCH group, ^Δ^*p* < 0.05.

The FBG and HbA1c of the OH group and the SCH group were higher than the low T_4_ group and the normal group, and the OH group was higher than the SCH group (*p* < 0.05), as shown in Figure 1.

### Comparison of Blood Lipid Levels in Each Group

**Figure 2:**
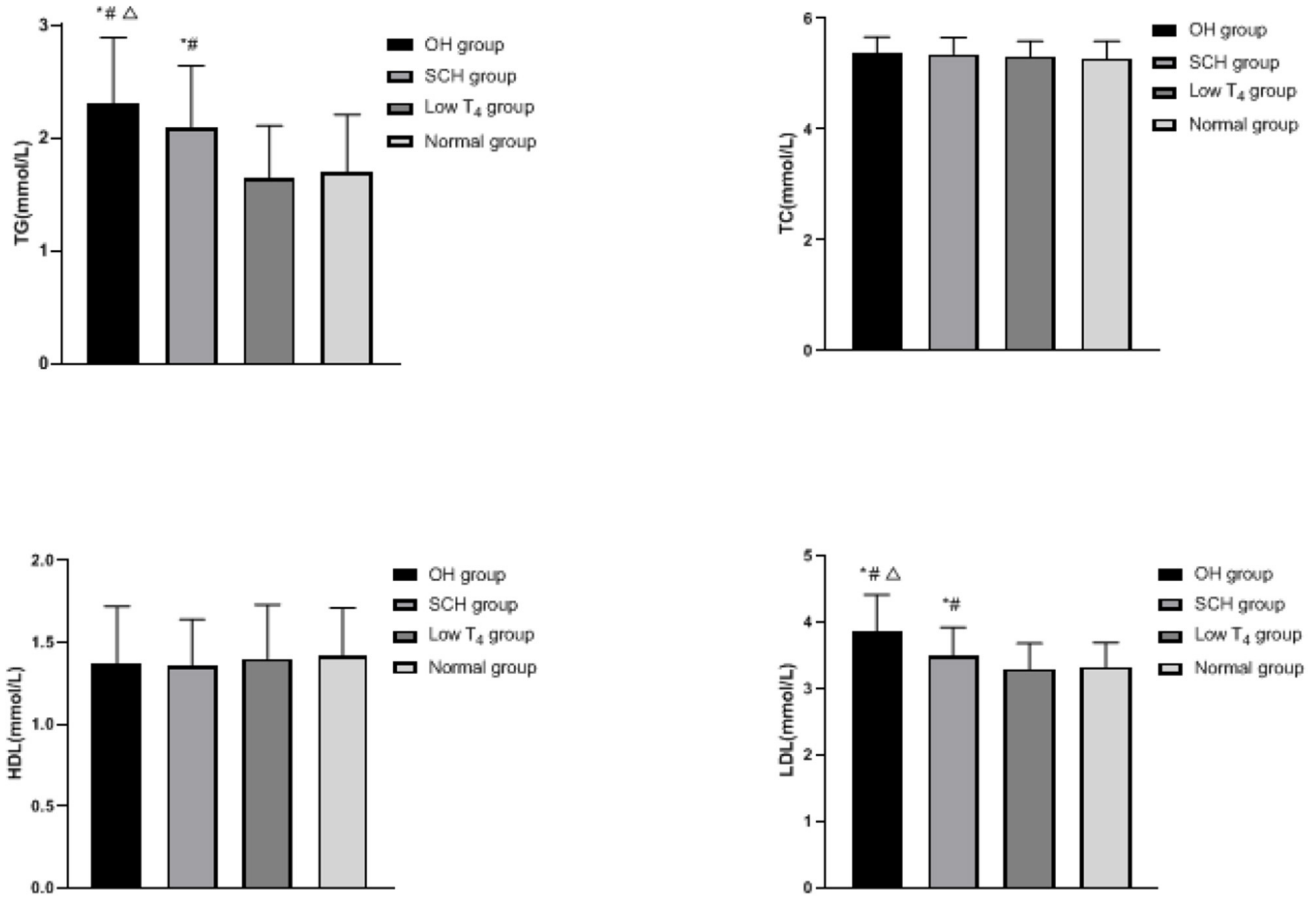
Comparison of blood lipid levels in each group: compared with the normal group, ^*^p < 0.05; compared with the low T_4_ group, ^#^*p* < 0.05; compared with the SCH group, ^Δ^*p* < 0.05.

The TG and LDL of the OH group and the SCH group were higher than the low T_4_ group and the normal group, and the OH group was higher than the SCH group (*p* < 0.05), as shown in Figure 2.

### Comparison of Adverse Pregnancy Outcomes Between the Two Groups

The incidence of premature delivery and PROM at term in the hypothyroidism non-control group was higher than the hypothyroidism control group (*p* < 0.05), as shown in Table 1.

**Table 1:** Comparison of adverse pregnancy outcomes between the two groups (*n*,%).

**Group**	**Premature delivery**	**Abortion**	**PROM at term**	**Infant of low-birth weight**	**Fetal distress**
Hypothyroidism non-control	3 (12.50%)	2 (8.33%)	5 (20.83%)	1 (4.17%)	3 (12.50%)
group (*n* = 24)					
Hypothyroidism control	2 (2.35%)	2 (2.35%)	6 (7.06%)	3 (3.53%)	7 (8.24%)
group (*n* = 85)					
*χ^2^*value	4.403	1.894	3.914	0.021	0.409
*P*-value	0.036	0.169	0.048	0.883	0.523

### Comparison of Fetal Growth and Development Between the Two Groups

**Figure 3:**
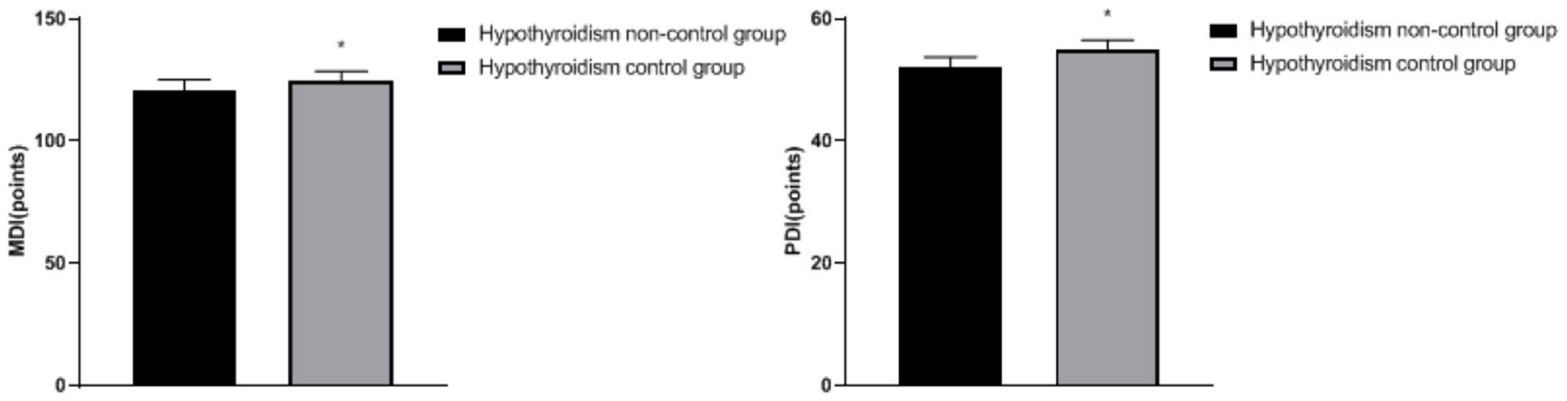
Comparison of fetal growth and development between the two groups: compared with the hypothyroidism non-control group, ^*^p < 0.05.

The MDI and PDI in the hypothyroidism non-control group were lower than the hypothyroidism control group (*p* < 0.05), as shown in Figure 3.

## Discussion

Studies have shown that severe thyroid dysfunction is closely related to female infertility, poor pregnancy outcomes, and offspring stunting (9). At present, during pregnancy, thyroid function can be evaluated by detecting serum FT_3_, FT_4_, and TSH levels. Some scholars believe that hypothyroidism in early pregnancy does not increase the risk of dysglycemia and dyslipidemia, while the levels of FT_4_ and TSH in the second trimester are related to glucose and lipid metabolism (10). Therefore, by detecting the levels of FT_3_, FT_4_, and TSH in pregnant women in the second trimester, we divided the patients with hypothyroidism into the OH group, the SCH group, and the low T_4_ group, observed the glucose and lipid metabolism in pregnant women, and discussed the influence of hypothyroidism on a pregnancy outcome and fetal growth and development.

In this study, FBG, HbA1c, TG, and LDL in the OH group and the SCH group are higher than the low T_4_ group and the normal group, and the increase in the OH group is more significant, which indicates that pregnant women with hypothyroidism during pregnancy are more prone to glucose and lipid metabolism disorder. TH has a two-way characteristic in regulating glucose metabolism of the body. On the one hand, it can increase blood glucose by promoting the decomposition and utilization of glycogen, enhancing gluconeogenesis and increasing the rate of glucose metabolism. On the other hand, it can increase glycolysis by increasing insulin secretion, thereby reducing blood glucose (11, 12). For patients with hypothyroidism, most patients will have positive thyroid peroxidase antibody, TH decreased and abnormal immune function, which will further affect the utilization of insulin in peripheral tissues. The phenomenon of insulin resistance in pregnant women will lead to abnormal glucose metabolism and promote the change of fasting glucose tolerance, and thus lead to the increase of blood glucose (13). Jia's team found that maternal glucose metabolism during pregnancy was more sensitive to changes of thyroid hormone than that during non-pregnancy, and the incidence of gestational diabetes in pregnant women with hypothyroidism was higher than that in pregnant women with normal thyroid function (14). In addition, hypothyroidism is also closely related to the fat metabolism of pregnant women. The main reasons are as follows: (1) TH has influence on the fat synthesis, transportation, and degradation. Compared with the normal population, the cholesterol transport level in the patients with hypothyroidism decreased, the carrying capacity of apolipoprotein to TG and LDL was affected, and the activity of lipoprotein decreased, resulting in the decrease of cholesterol clearance and degradation, so the concentration of TG and LDL in blood increased. (2) By regulating the expression of the LDL receptor and the activity of lipoprotein lipase on the surface of liver cells, the occurrence of hypothyroidism can interfere with the reverse transport process of cholesterol in liver epithelial cells by promoting the occurrence of oxidative stress disorder *in vivo*, and lead to the decrease of LDL receptor sensitivity, the decrease of the number and activity of the LDL receptor on the surface of liver cells, the decrease of LDL clearance and degradation, the accumulation of LDL, and the abnormality of serum lipid metabolism. (3) Hypothyroidism will cause a large amount of free fatty acids to flow into the liver, which will increase the synthesis of LDL in the liver, and eventually lead to the increase of TG and LDL content (15–17).

This study also found that treatment with levothyroxine sodium tablets significantly improved thyroid function of patients with hypothyroidism during pregnancy, and the risk of premature delivery and PROM was lower. Without treatment for hypothyroidism during pregnancy, the metabolism of blood glucose and blood lipid may be disordered, and TH is directly involved in placenta development, and OH or SCH may lead to mild deficiency of TH, which may lead to premature birth of newborn (18). At the same time, due to the influence of gestational diabetes mellitus, lower genital tract infection, mechanical stimulation, the increase of interleukin-6 (IL-6) and tumor necrosis factor-α (TNF-α) cytokines, and other factors, PROM at term has become one of the clinical adverse pregnancy outcomes (19). Adipose is involved in the energy metabolism of pregnant and lying-in women. IL-6 and TNF-α are endocrine hormones secreted by adipose tissue. The serum of TSH can regulate the endocrine function of adipose tissue to a certain extent (20). However, in the body of pregnant women with OH or SCH, the serum TSH level is obviously increased, which can promote the secretion of leptin, adiponectin, IL-6, TNF-α, and other inflammatory factors in adipose tissue, leading to PROM at term (21).

In addition, TH not only affects the proliferation and migration of neurons in cerebral cortex, and the uplift of hippocampus and inner pleural ganglion but also influences the formation of axons and dendrites, and myelination (22). During pregnancy, the fetus needs TH to ensure the development of normal nervous system and other organ systems, and TH plays a key role in the development and maturation of the fetal brain (23). Once hypothyroidism occurs during pregnancy, the increase of TSH level may inhibit the secretion of human chorionic gonadotropin by placenta to a certain extent, resulting in an irreversible influence on the development of placenta and fetus and damages the development of fetal nervous system (24). Moreover, long-term hypothyroidism will make the abnormal blood glucose and blood lipid levels of pregnant women for a long time, resulting in the damage of the blood vessel wall and the decrease of blood flow supply, which will further lead to the decrease of blood flow to various organs of the body, resulting in the lack of oxygen supply to cells, and, in severe cases, placenta aging will occur, resulting in the limitation of fetal growth and development (25). The above research is consistent with our results. We have observed that, after hypothyroidism control, infants' intelligence and psychomotor development are better.

## Conclusion

To sum up, pregnant women with hypothyroidism during pregnancy are more prone to glucose and lipid metabolism disorder, which increases the risk of premature delivery and PROM at term, and has certain influence on the intellectual development and psychomotor development of infants. The results suggest that doctors should screen thyroid function during pregnancy, pay attention to the changes of glucose and lipid metabolism indexes of pregnant women with hypothyroidism, and actively give treatment according to the actual situation of patients so as to improve the maternal and child outcomes.

## Data Availability Statement

The original contributions presented in the study are included in the article/supplementary material, further inquiries can be directed to the corresponding author.

## Ethics Statement

The studies involving human participants were reviewed and approved by the Ethics Committee of the Zhuji People's Hospital. The patients/participants provided their written informed consent to participate in this study.

## Author Contributions

All authors of this study made equal contributions, including study design, inclusion of cases, data detection and statistics, and writing of the paper. HZ was the supervisor of the entire study. All authors contributed to the article and approved the submitted version.

## Conflict of Interest

The authors declare that the research was conducted in the absence of any commercial or financial relationships that could be construed as a potential conflict of interest.

## Publisher's Note

All claims expressed in this article are solely those of the authors and do not necessarily represent those of their affiliated organizations, or those of the publisher, the editors and the reviewers. Any product that may be evaluated in this article, or claim that may be made by its manufacturer, is not guaranteed or endorsed by the publisher.
